# The impact of mobile internet usage patterns on employment intentions of medical students: A cross-sectional study

**DOI:** 10.1371/journal.pone.0340254

**Published:** 2026-01-21

**Authors:** Jiajing Hu, Nan Zhang, Limin Duan, Xiaodong Ren

**Affiliations:** Inner Mongolia Medical University, Hohhot, China; Penn State University: The Pennsylvania State University, UNITED STATES OF AMERICA

## Abstract

**Background:**

China’s healthcare system is undergoing major transformations, with urbanization worsening the unequal distribution of medical resources and primary healthcare institutions facing persistent workforce shortages. Meanwhile, medical students must choose between further education and entering the workforce. The widespread use of mobile internet has reshaped career decision-making, yet its impact on medical students’ employment intentions (EI) and primary care employment intentions (PCEI) remains unclear. Previous studies have focused on traditional factors, such as sociodemographic characteristics and academic experiences, but research on how mobile internet usage intensity and types influence career choices is limited. This study examines the relationship between mobile internet use and EI/PCEI, providing empirical insights into the role of digital engagement in career decisions.

**Methods:**

A cross-sectional survey was conducted among undergraduate medical students at Inner Mongolia Medical University in May–June 2024. Data were collected via an online questionnaire assessing sociodemographic factors, mobile internet usage intensity and type, and EI. Multiple linear regression models were used to analyze associations between mobile internet use and EI, adjusting for sociodemographic variables.

**Results:**

A total of 4,494 valid responses were analyzed. Higher mobile internet usage intensity was significantly associated with lower employment intention (EI) and primary care employment intention (PCEI). Compared with students using mobile internet for less than 1 hour per day, those using it for 1–3 hours showed significantly lower EI (β = −0.80, OR = 0.45) and PCEI (β = −0.75, OR = 0.47), with the strongest negative associations observed among students using mobile internet for more than 5 hours per day. Regarding usage type, students primarily engaged in leisure and entertainment or social networking reported significantly lower EI and PCEI than those using mobile internet mainly for academic and professional development, whereas e-commerce and lifestyle services showed no significant associations. Rural background was positively associated with PCEI, while higher academic year was associated with lower EI and PCEI.

**Conclusions:**

Higher mobile internet usage intensity and non-academic usage patterns are associated with lower employment intention, particularly reduced willingness to enter primary care. These findings highlight the need for policy and practice-oriented interventions, including integrating digital self-regulation and critical digital literacy into medical education, as well as strengthening positive digital representations of primary care careers. Such measures may support more informed career decision-making and contribute to workforce planning in underserved regions.

## Introduction

The rapid development of mobile internet has profoundly impacted the global economy [1], social structures [2,3], and education systems [4]. As a core carrier of information technology, mobile internet has not only driven the growth of the digital economy but has also significantly transformed individuals’ learning patterns, career development, and social interactions [5–7]. In higher education, the widespread adoption of mobile internet has provided university students with extensive learning resources and career development opportunities while subtly shaping their employment perceptions and career decision-making [8]. With ongoing technological and social transformations, career intentions are increasingly influenced not only by traditional factors, but also by patterns of digital engagement, particularly through mobile internet use.

According to data from the China Internet Network Information Center (CINIC), the number of mobile Internet users in China had reached 1.09 billion in 2023 [9]. Among younger demographics, mobile internet has become an essential platform for learning [10,11], social networking, and job searching [12]. While it facilitates greater access to information, its intensity of use and content engagement patterns may also prolong decision-making and reshape career expectations, particularly in competitive fields such as medicine. The continuous expansion of mobile communication systems further highlights the complexity of digital ecosystems in which students engage [13–15].

In the Chinese higher education system, medical education follows a standardized five-year undergraduate curriculum, leading to a Bachelor of Medicine degree (equivalent to MBBS). Upon graduation, students may pursue postgraduate studies (residency or master’s degrees), enter clinical practice (typically in hospitals), engage in research, or work in primary healthcare institutions such as township health centers or community clinics. Career choices are shaped by a combination of individual aspirations, institutional exposure, and healthcare system demands. [16–18].

Inner Mongolia, where the current study was conducted, is a large, ethnically diverse, and economically developing region in northern China [19]. It faces structural healthcare challenges, including a chronic shortage of medical personnel in rural and pastoral areas, which makes medical graduates’ career decisions particularly consequential to regional healthcare delivery [20]. Moreover, medical education in Inner Mongolia is subject to national standards, but students often encounter region-specific exposure to primary care environments, which may influence their employment intentions differently from those in more urbanized provinces.

In recent years, the Chinese government has prioritized the development of primary care systems through major national initiatives such as the Healthy China 2030 Plan and the Opinions on Deepening the Integration of Medical Education. These policies advocate for the expansion of grassroots healthcare services, including general practice and township-level care, and emphasize strengthening the training and placement of medical graduates in underserved areas. However, despite policy incentives (e.g., tuition waivers, guaranteed placements), primary care positions continue to face low uptake among medical graduates [21,22]. This disparity is not unique to China but is commonly observed in health systems worldwide [23]. In this study, we define “primary care employment intentions (PCEI)” as the willingness to work in community-level or rural health institutions, consistent with the policy goal of “strengthening grassroots healthcare.”

Employment intentions (EI) refer to medical students’ general willingness to enter the workforce after graduation, regardless of clinical setting or specialty. While medical graduates in China face diverse career options—including further study, research, and various forms of clinical work—our study focuses on EI and PCEI because these outcomes are directly tied to current healthcare system reform priorities and medical workforce planning.

Medical students’ EI is shaped by a complex interaction of personal characteristics, institutional contexts, and national health policies. Prior studies have shown that sociodemographic variables (e.g., gender, rural/urban origin, family background), clinical exposure, and perceptions of workload, prestige, and income significantly influence career choices [24–26]. For instance, students from rural backgrounds are more likely to express interest in primary care, especially when supported by targeted financial or educational policies [27,28]. Emerging evidence also suggests that digital content environments can significantly shape individuals’ perceptions, preferences, and decision-making processes, indicating that digital experiences may influence career intentions in similarly complex ways [29]. However, despite these findings, there remains a lack of empirical research investigating how digital behavior affects EI and PCEI.

Unlike existing studies that primarily examine social media use or general internet exposure in relation to employment outcomes, this study advances the literature in three key ways. First, it systematically distinguishes both the intensity and functional types of mobile internet usage, allowing a more nuanced understanding of digital behavior. Second, it focuses on medical students, a group facing highly structured career pathways and policy-driven workforce expectations, yet largely overlooked in digital behavior research. this study analyze the situation in Inner Mongolia, a region with underdeveloped services and a unique culture, and discuss how regional context and digital participation work together to influence employment intentions.

**Fig 1:**
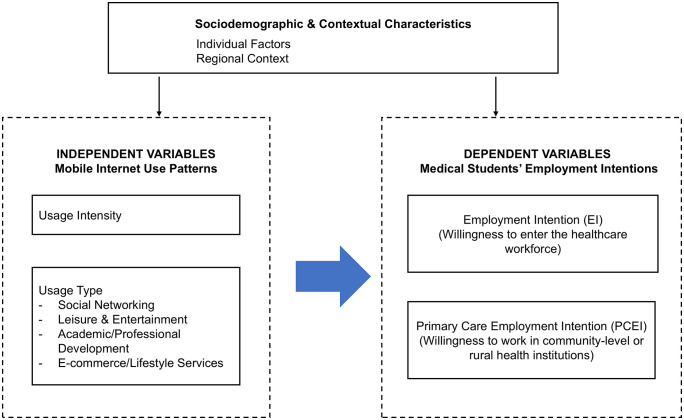
Conceptual framework linking mobile internet use to medical students’ employment intentions.

To guide the analytical approach of this study, Fig 1 presents a conceptual framework illustrating the proposed relationships between mobile internet use patterns and medical students’ employment intentions. The framework situates mobile internet usage as the key independent variables, while the two dimensions of employment intentions serve as the dependent outcomes. Sociodemographic and contextual characteristics, including individual background factors and the regional context of Inner Mongolia, are incorporated as covariates that may shape both digital behavior and career preferences. This framework reflects the assumption that mobile internet usage patterns within broader structural and cultural contexts, influencing students’ perceptions of the healthcare profession and their willingness to enter the workforce or pursue primary care roles.

To address this research gap, our study aims to explore the following key questions: (1) Does the intensity of mobile internet usage influence medical students’ EI and PCEI? (2) Do different types of mobile internet usage—such as social interaction, consumption and lifestyle services, leisure and entertainment, and academic and professional development—exert differential effects on EI and PCEI?

To explore these questions, we conducted a cross-sectional survey among undergraduate medical students at Inner Mongolia Medical University, analyzing the impact of mobile internet usage intensity and patterns on employment decision-making.

## Methods

### Study design

This cross-sectional single-institution study was conducted among undergraduate medical students at Inner Mongolia Medical University to analyze their employment intentions, mobile internet usage patterns, and associated factors. The study was approved by the Ethics Committee of Inner Mongolia Medical University (No. YKD202201083) and was conducted in strict accordance with the Declaration of Helsinki. All participants provided informed consent prior to participation. The authors did not have access to any personally identifiable information during or after data collection.

All data were collected through an online questionnaire, which was developed and reviewed by Professor Zhang Nan and her colleagues. Prior to the formal survey, the questionnaire was pilot-tested among a small group of undergraduate medical students to assess question comprehension, wording clarity, and survey flow. Minor revisions were made based on the pilot feedback. The main study was conducted between May and June 2024, and the survey was distributed via an online platform Wenjuanxing (http://www.wjx.com, Changsha Ranxing Information Technology Co., LTD, Changsha, China). The survey link and study information were disseminated through the instant messaging application WeChat, where participants were provided with detailed explanations of the study’s objectives and procedures.

Participants accessed the survey by scanning a QR code or clicking a URL link. Upon entering the survey, they were presented with an information sheet outlining the study’s purpose and content. Prior to participation, electronic informed consent was obtained from all respondents, and those who declined to participate were allowed to exit the survey at any time.

### Variables and measurement

To ensure participant privacy, this study was conducted anonymously. The questionnaire comprised three sections:

1) Sociodemographic characteristics, including gender, grade, ethnicity, political affiliation, family economic hardship, place of residence, and medical background in the family.
2) Mobile internet engagement, measured in terms of usage intensity and main usage type. Usage intensity was assessed based on the average daily time spent on mobile internet on non-holiday weekdays, with response options: < 1 hour, 1–3 hours, 3–5 hours, and >5 hours. Usage type was categorized into social interaction, consumption and lifestyle services, leisure and entertainment, and academic and professional development. Participants were asked to select the primary type of mobile internet engagement that best represented their usage pattern.
3) Employment intention, which measuring participants’ post-graduation employment intentions and willingness to work in primary care settings. Responses were recorded on a five-point Likert scale: 1 (strongly unwilling), 2 (unwilling), 3 (neutral), 4 (willing), and 5 (strongly willing).

### Data collection and quality control

The survey was conducted over a period of three weeks. Initially, researchers contacted counselors from each academic department to explain the study’s purpose and procedures. The counselors then distributed the survey QR code and link to eligible participants. After all samples are submitted, an Excel file is generated for statistical analysis.

To ensure data integrity and exclude non-medical students, Participants whose IP addresses were outside the city where Inner Mongolia Medical University is located (n = 16) were removed, responses from non-medical majors (n = 37) were considered invalid and excluded, responses from graduate students (n = 6) were also excluded. In addition, duplicate submissions were identified using IP checks and manual inspection of suspiciously similar response patterns to ensure data authenticity and validity.

After applying these exclusion criteria, the final valid sample size consisted of 4,494 participants, yielding a response validity rate of 98.7%.

### Data analysis

All statistical analyses were conducted using Stata 17.0 (Stata Corp LLC, College Station, TX, USA). Descriptive statistics were used to summarize the demographic characteristics and key study variables.

Given that both EI and PCEI were measured on 5-point Likert scales, we employed ordered logistic regression models as the primary analytical approach. The ordered logit model is appropriate for ordinal outcomes because it preserves their rank-order structure without assuming equal intervals between response categories. All statistical tests were two-tailed, with significance levels set at 0.05, 0.01, and 0.001.

## Results

### Descriptive statistics

Among the 4,494 undergraduate medical students surveyed, 64.66% were female, and 35.34% were male. 54.54% of students came from rural areas, while 45.46% were from urban areas. 41.41% were from ethnic minority groups.

Students were distributed across different academic years, with the highest proportion in Grade 2 (30.17%), followed by Grade 1 (27.21%) and Grade 3 (23.34%), while Grades 4 and 5 accounted for 10.64% and 8.63%, respectively. 71.43% of students were members of the Communist Youth League. 34.96% of students reported family economic hardship, and 29.57% had family members working in the medical field. The demographic characteristics of the subjects who completed the questionnaires are shown in S1 Table.

In terms of mobile internet usage intensity, 37.56% of students used mobile internet for 3–5 hours per day, followed by 32.53% for 1–3 hours per day, while 23.65% reported using it for more than 5 hours per day, and 6.25% for less than 1 hour per day. Regarding primary mobile internet activities, 45.04% of students mainly used mobile internet for leisure and entertainment, while 30.49% used it for educational and professional development. 21.96% reported social networking as their primary activity, and 2.51% used it mainly for e-commerce and lifestyle services.

The mean EI score was 3.15 ± 1.28, while the mean PCEI score was 3.05 ± 1.23, indicating a moderate level of preference for employment and a slightly lower inclination toward primary care roles.

### Regression analysis

This section presents the results of the multiple linear regression models examining the relationship between mobile internet usage and EI and PCEI of undergraduate medical students. Mobile internet usage intensity and main mobile internet usage type were included in the model as key independent variables, while controlling for demographic characteristics.

Before estimating the regression models, we examined potential multicollinearity among the variables using Spearman correlation coefficients (see S2 Table). The results showed that the correlations among all variables were generally low, with none exceeding commonly accepted thresholds (r > 0.70). The highest correlation was observed between EI and PCEI (r = 0.676), which is expected given their conceptual proximity, but this does not affect the independent variable structure of the models. Mobile internet usage intensity and main usage type showed weak correlations with each other and with sociodemographic controls, indicating no risks of redundancy or instability in parameter estimation. Overall, the correlation matrix suggests that multicollinearity is unlikely to bias the regression results, and all variables were retained in subsequent analyses.

#### Effect of mobile internet usage intensity on employment intentions.

Table 1 reports the estimated coefficients for mobile internet usage intensity, using students with less than 1 hour of daily usage as the reference group. The results indicate a negative association between higher internet usage intensity and EI. Specifically, compared to students with minimal mobile internet usage, those who use mobile internet for 1−3 hours per day exhibit significantly lower EI (*β* = −0.8017, *p* < 0.01, *OR*=0.4486) and PCEI (*β* = −0.7454, *p* < 0.01, *OR*=0.4745). These *OR* suggest that students in the 1–3 hours group are about 55% less likely to express stronger overall employment intention and about 53% less likely to express stronger primary care employment intention than those with minimal usage.

**Table 1:** Regression results for the effects of mobile internet usage on employment intentions.

	EI (β, SE)	PCEI (β, SE)
**Mobile Internet Usage Intensity (Ref: < 1h/day)**		
1–3 h/day	−0.8017*** (0.1244)	−0.7454*** (0.1259)
3–5 h/day	−0.8958*** (0.1243)	−0.9279*** (0.1258)
> 5 h/day	−1.0221*** (0.1304)	−1.1225*** (0.1321)
**Main Mobile Internet Usage Type (Ref: Educational & Professional Development)**		
Social Networking	−0.2397*** (0.0757)	−0.1564** (0.0763)
E-commerce & Lifestyle Services	0.0305 (0.1747)	−0.1111 (0.1777)
Leisure & Entertainment	−0.2087*** (0.0651)	−0.2849*** (0.0660)
**Gender**	−0.0737 (0.0573)	−0.0927 (0.0578)
**Residence**	0.2725*** (0.0569)	0.2748*** (0.0571)
**Ethnicity**	0.1453*** (0.0467)	0.0522 (0.0471)
**Grade**	−0.0358 (0.0240)	−0.0918*** (0.0241)
**Political Affiliation**	0.0726 (0.0573)	−0.0034 (0.0578)
**Economic Hardship**	−0.0548 (0.0586)	−0.0941 (0.0589)
**Family Medical Bacnground**	−0.0046 (0.0608)	−0.0092 (0.0614)
***R*** ^***2***^	0.0101	0.0127
***Likelihood Ratio χ²***	141.5000	175.0200

Standard errors are reported in parentheses. Significance levels: *p < 0.1, **p < 0.05, ***p < 0.01.

The negative association becomes stronger with increasing usage intensity, with the largest reduction observed in the > 5 hours per day group (*β* = −1.0021, *p* < 0.01, *OR*=0.3598 for EI; *β* = −1.1225, *p* < 0.01, *OR*=0.3255 for PCEI). These results indicate that students with high mobile internet use are 63% less likely to report stronger EI and 67% less likely to report stronger PCEI than students who use it for less than 1 hour per day. Taken together, these findings suggest that prolonged mobile internet usage is associated with a marked decline in medical students’ employment aspirations, particularly in relation to primary care roles.

#### Effect of mobile internet usage type on employment intentions.

Regarding main mobile internet usage type, the results demonstrate significant differences depending on students’ primary internet activities, with educational and professional development serving as the reference category. Students who mainly use mobile internet for social networking report significantly lower EI (*β* = −0.2397, *p* < 0.01, *OR*=0.7868) and a marginally lower PCEI (*β* = −0.1564, *p* < 0.05, *OR*=0.8552). The social networking–oriented users are approximately 20% less likely to express stronger EI than students who use mobile internet for academic or career-related purposes. Although the magnitude for primary care intention is smaller, the downward shift remains meaningful, suggesting that socially driven digital engagement may dilute career focus.

Similarly, students whose primary mobile internet use is leisure and entertainment exhibit significantly reduced EI (*β* = −0.2087, *p* < 0.01, *OR*=0.8116) and PCEI (*β* = −0.2849, *p* < 0.01, *OR*=0.7521). These results suggest that leisure-oriented users are 19% less likely to report stronger overall employment intention and 25% less likely to express stronger primary care intention, relative to academically oriented users. The stronger reduction in primary care intention implies that leisure-focused digital consumption may be particularly misaligned with the values, expectations, and work characteristics associated with primary care careers.

In contrast, students who primarily engage in e-commerce and lifestyle services do not show statistically significant differences in EI compared to those who use mobile internet for academic and career-related purposes. These results suggest that mobile internet activities related to leisure and social interactions may negatively impact students’ career aspirations, while educationally oriented usage is more conducive to maintaining strong EI.

#### Effects of control variables.

Among the control variables, residence and ethnicity emerge as significant predictors of EI. Students from rural backgrounds report significantly higher EI (β = 0.2725, p < 0.01) and PCEI (β = 0.2748, p < 0.01), suggesting that rural-origin students may have stronger motivations to secure employment. Additionally, students from ethnic minority groups report higher overall EI (β = 0.1453, p < 0.001), though the effect is not significant for PCEI.

Grade level is negatively associated with PCEI (β = −0.0918, p < 0.01), indicating that as students’ progress through medical school, their employment aspirations may decline, particularly regarding primary care careers. Other control variables, including gender, political affiliation, family economic hardship, and family medical background, do not exhibit statistically significant effects on EI.

### Robustness analysis

To evaluate the robustness of the primary ordered logistic regression results, two supplementary analytical strategies were conducted. First, ordinary least squares (OLS) regressions were estimated by treating EI and PCEI as continuous variables. Second, EI and PCEI were redefined as binary variables by excluding respondents who selected the neutral category (score of 3) and recoding scores of 1–2 as low intention and 4–5 as high intention. Binary logistic regression models were then estimated to further validate the direction and significance of the associations. The results are presented in Table 2.

**Table 2:** Robustness analysis results.

	OLS (EI)	OLS (PCEI)	Logit (Binary EI)	Logit (Binary PCEI)
**Mobile Internet Usage Intensity (Ref: < 1h/day)**				
1–3 h/day	−0.4671*** (0.0830)	−0.4197*** (0.0793)	−0.8032*** (0.1764)	−0.6451*** (0.1736)
3–5 h/day	−0.5310*** (0.0831)	−0.5458*** (0.0794)	−0.9310*** (0.1768)	−0.8843*** (0.1738)
> 5 h/day	−0.6280*** (0.0873)	−0.6730*** (0.0834)	−1.1081*** (0.1845)	−1.1742*** (0.1823)
**Main Mobile Internet Usage Type (Ref: Educational & Professional Development)**				
Social Networking	−0.1500*** (0.0532)	−0.0932* (0.0508)	−0.2985*** (0.1038)	−0.2038* (0.1075)
E-commerce & Lifestyle Services	0.0500 (0.1241)	−0.0553 (0.1186)	0.2263 (0.2654)	−0.0850 (0.2593)
Leisure & Entertainment	−0.1290*** (0.0457)	−0.1782*** (0.0436)	−0.1521* (0.0895)	−0.3370*** (0.0919)
**Control Variables**	Included	Included	Included	Included
***R*** ^***2***^	0.0287	0.0363	0.0281	0.0338
***N***	4494	4494	3085	2838

Standard errors are reported in parentheses. Significance levels: *p < 0.1, **p < 0.05, ***p < 0.01.

The OLS results are consistent with the main ordered logit findings. Higher mobile internet usage intensity is significantly associated with lower EI and PCEI, with the strongest negative effects observed among students using mobile internet for more than 5 hours per day (p < 0.001). Similarly, students who mainly use mobile internet for social networking and leisure activities report significantly lower EI (p < 0.01), while those engaging in e-commerce and lifestyle services do not exhibit significant differences from the reference group.

The binary logistic regression results further confirm the robustness of the findings. The negative impact of higher mobile internet usage intensity remains statistically significant, and the effects of social networking and leisure-oriented internet usage continue to be associated with lower EI and PCEI (p < 0.05 or lower).

Overall, the robustness checks indicate that the main findings are not sensitive to model specification or the measurement of the dependent variables. The consistent results across different estimation methods reinforce the conclusion that higher mobile internet usage, particularly for non-academic purposes, is negatively associated with EI and PCEI.

## Discussion and conclusion

### Discussion

The rapid integration of mobile internet into daily life has profoundly influenced the structure of employment markets and transformed career decision-making processes across various industries [3,8]. This study provides new insights into the relationship between mobile internet usage patterns and career aspirations among undergraduate medical students in China, with a particular focus on EI and PCEI. Conducted within the unique sociocultural and geographical context of Inner Mongolia, this research adds an important layer of understanding regarding how regional characteristics interact with digital behaviors. Our findings reveal significant associations between mobile internet usage patterns, EI and PCEI, while also highlighting the role of demographic and contextual factors in shaping these outcomes.

A significant negative correlation was observed between high-intensity mobile internet use and both EI and PCEI, suggesting a possible negative correlation between digital connectivity and career expectations. This finding supports the Digital Saturation Hypothesis, which posits that exceeding cognitive thresholds in screen time may impair career decision-making efficiency [30]. These findings partially diverge from existing studies conducted among general college student populations. Prior research has reported a positive association between social media use and employment choices [31,32]. In contrast, our study identifies a predominantly negative association between high-intensity mobile internet use and employment intentions among medical students. This discrepancy may reflect differences in professional training intensity, career structures, and the nature of decision-making in medical education, where career choices are more constrained, long-term, and institutionally regulated. The vast territory and relatively dispersed population of Inner Mongolia can lead to a reliance on digital connectivity for information and social interaction, potentially increasing the risk of cognitive overload from high-intensity use. Additionally, the local culture highly values direct interpersonal connections. The rapid pace of digitalization, which can displace offline, community-oriented activities important for professional socialization, may therefore create a pronounced sense of dissonance. On the one hand, high-intensity mobile internet usage may displace essential career planning activities, such as clinical skill development and mentorship engagement, leading to inadequate career preparedness [33]. On the other hand, frequent switching between social, entertainment, and educational contexts may reduce the ability to integrate information deeply [34], which is important for medical students’ career decision-making, as it requires the synthesis of multiple factors, including policy interpretation and community healthcare needs.

Notably, the decline in PCEI was more pronounced than that in EI, suggesting a higher cognitive load sensitivity in primary care career decisions. This is particularly salient in Inner Mongolia, where primary care roles often entail serving in remote banners (counties) or pastoral areas, requiring an understanding of unique challenges like geographical barriers and higher prevalence of certain chronic diseases. Compared to specialty careers, primary care career decisions require a balance between economic incentives, social prestige, and policy-driven motivations [35–37]. The assessment of this balance relies more heavily on coherent information processing, making it more susceptible to cognitive overload [38]. Excessive digital engagement may undermine high-order cognitive functions [39], leading students to rely more on intuition or societal stereotypes (e.g., primary care is equal to low technical competency) in their decision-making.

The differential impact of various mobile internet usage types suggests behavioral heterogeneity, an aspect often overlooked in existing research on digital engagement and employment intentions. Our findings suggest that different types of mobile internet use have different effects on medical students’ EI and PCEI. Using educational and professional development-related usage as the reference category, we observed that leisure and entertainment use and social networking use were significantly associated with lower employment intentions, whereas e-commerce and lifestyle services usage showed no significant effect.

The negative correlation between leisure and entertainment usage and EI, particularly PCEI, suggests that such digital engagement may disrupt career planning and reduce motivation for medical practice. One possible explanation is that immersive entertainment provides immediate gratification [40], which over time may inhibit motivation for long-term career planning [41]. Furthermore, given the high academic and psychological pressures faced by medical students, gaming and entertainment may be used as coping mechanisms for stress relief, potentially creating a negative cycle of stress-avoidance-career disengagement [42]. This effect is particularly pronounced in primary care career decision-making, as primary care roles are often associated with challenging working conditions and lower financial returns.

The negative association between social networking usage and EI suggests that, while digital social platforms facilitate career-related interactions, they may also have detrimental effects. Social media content distribution algorithms tend to prioritize narratives of specialist doctors’ achievements (e.g., high-impact publications, prestigious conference presentations) [43], lead to the marginalization of primary care physicians’ professional narratives [44]. This “visibility imbalance” systematically undermines the perceived value of primary care careers. Medical students frequently evaluate their own career trajectories by comparing themselves to peers, mentors, or professionals encountered on digital platforms. This process may lead to a sense of relative deprivation, fostering avoidance emotions toward certain career paths [45]. Additionally, social networking may encourage passive career engagement, where students consume career-related content without actively participating in job-seeking, professional networking, or skill development. The distinction between active and passive digital engagement is critical—while professional engagement via social media can enhance career preparedness, superficial or purely social interactions appear to diminish career readiness [46].

Unlike leisure and social networking activities, engagement in e-commerce and lifestyle services did not significantly influence EI or PCEI. This suggests that such activities are primarily transactional and utilitarian [47], fulfilling specific needs rather than influencing long-term career aspirations. Unlike entertainment and social networking, which shape attitudes, aspirations, and perceptions through prolonged engagement, e-commerce interactions are typically goal-oriented and short-lived, making them less likely to influence students’ career-related cognition. However, the growing integration of digital health services, such as telemedicine platforms, online pharmaceutical services, and healthcare-related e-commerce, raises important questions about whether different forms of lifestyle-related digital engagement may shape future career preferences. For instance, students who frequently use digital healthcare services may gain greater exposure to primary care models, patient interactions, or entrepreneurial opportunities, subtly shaping their career outlook. Future research should explore how specific forms of digital engagement within e-commerce platforms may influence medical career preferences over time.

Demographic factors, particularly place of origin and academic year, significantly moderated EI and PCEI, reflecting the influence of policy environments and clinical experiences on career decision-making. Rural-origin medical students demonstrated higher EI and PCEI than their urban counterparts, likely due to a combination of policy-driven incentives and pragmatic family decision-making cultures [28]. Policy commitment effects, such as tuition waivers and guaranteed job placements for rural medical students, reduce uncertainty regarding career prospects, thereby increasing their willingness to pursue employment [48]. Additionally, rural families tend to prioritize employment stability [49,50], and this intergenerational transmission of career values may further encourage students to choose primary care positions, which offer greater job security.

Moreover, the grade level was negatively associated with EI, aligning with the Erosion of Idealism Theory [51]. Clinical internships likely provide a more realistic and demanding perspective on medical practice, exposing students to the high levels of stress and challenges associated with healthcare work [52]. Furthermore, first-hand experiences of resource constraints in primary care settings and limited career advancement opportunities may contribute to a decline in enthusiasm for medical practice, particularly in primary healthcare roles. In Inner Mongolia, these effects may be further amplified by the region’s uneven distribution of healthcare resources, where clinical rotations may increase the likelihood that students are placed in rural and pastoral areas with significant understaffing and limited medical infrastructure. Additionally, the cultural expectation to serve local communities, coupled with limited pathways for professional development in county- and township-level institutions, may intensify the erosion of idealism as progress through training.

Overall, we recommend a multi-level intervention framework to mitigate the impact of mobile internet use on career decision-making.

Firstly, universities can encourage students to use existing tools such as Digital Wellbeing (Android) or Screen Time (iOS), which are commonly integrated into smartphones and allow users to monitor their app usage and receive non-intrusive reminders. Guidance on digital self-regulation can be incorporated directly into career planning curricula, helping students reflect on how their mobile internet usage patterns may influence time management and long-term career development. This approach aligns with adult learning principles by preserving students’ autonomy while promoting critical digital literacy and intentional technology use. Rather than imposing restrictions, it fosters reflective behaviors that support more informed and goal-oriented career planning.

Secondly, to address the systematic devaluation of primary care careers due to social media algorithmic bias, local governments could adopt strategies similar to the “Key Opinion Leader (KOL) Interventions” [53,54]. Reshaping the digital career narrative could increase career the visibility and attractiveness [55]. One approach is to train primary care physicians to produce high-quality short-form video content (e.g., “Innovations in Rural Chronic Disease Management” or “Building Trust in Community Doctor-Patient Relationships”). In collaboration with regulatory authorities, social media platforms could be required to prioritize the exposure of such content in recommendation algorithms, increasing public awareness and shifting perceptions of primary care professions.

Finally, at the individual level, enhancing medical students’ digital literacy is important. Incorporating “critical digital literacy” training modules into medical curricula could teach students to decode the logic behind content recommendation algorithms, recognize implicit career biases, and resist algorithmic manipulation that reinforces occupational stereotypes.

This study makes several contributions to the existing literature. Theoretically, it extends the application of the Digital Saturation Hypothesis and Erosion of Idealism Theory to the context of medical career decision-making, demonstrating how digital behaviors may interact with professional socialization processes. Methodologically, by treating employment intention and primary care employment intention as ordinal outcomes and applying ordered logistic regression, this study improves the analytical rigor. Practically, the findings offer policy-relevant insights for medical education and workforce planning in underserved regions, suggesting that digital engagement patterns should be considered alongside traditional incentives when designing interventions to promote primary care careers.

### Limitations

Several limitations that should be acknowledged in this study. First, the use of a cross-sectional design and self-reported data restricts causal inference. The observed associations between mobile internet usage patterns and employment intention cannot establish temporal ordering or rule out potential endogeneity. Self-reported mobile internet usage, including daily use duration and primary activity type, may be subject to recall bias, and future research would benefit from incorporating objective measures such as screen-time records or app usage logs to improve accuracy. Second, although exclusion criteria based on IP address and major/degree information were applied, duplicate responses may still occur.

Third, the categorization of mobile internet use intensity into four groups may oversimplify usage behaviors and reduce statistical sensitivity. Similarly, limiting respondents to a single “main usage type” may not fully capture multimodal online behaviors. Future studies could use continuous measures or multiple-choice/weighted usage indicators for greater precision.

Fourth, the explanatory power of the models is relatively low, as reflected by the small R² values. This suggests that important determinants, such as psychological characteristics, institutional factors, or family influences were not captured. Longitudinal or mixed-method designs are recommended to unpack complex causal pathways and contextualize behavioral mechanisms.

### Conclusion

This study elucidates the impact of mobile internet use on medical students’ EI and PCEI. While digital tools enhance access to information and professional networks, excessive or unstructured usage may negatively affect EI, particularly in relation to primary care roles.

Our findings provide practical insights for educators, policymakers, and students, enabling them to better navigate the intersection of technology and career development in the medical field. Moving forward, effectively aligning digital engagement with career preparedness will be essential in addressing healthcare workforce challenges.

## Supporting information

S1 Table Demographic characteristics of undergraduate medical students (N = 4494). (PDF)

S2 Table Spearman correlation matrix for variables used in regression models. (PDF)
