# Supplementary material for: The impact of mobile internet usage patterns on employment intentions of medical students: A cross-sectional study
Source: PLoS One. 2026 Jan 21;21(1):e0340254. doi: 10.1371/journal.pone.0340254 (PMC12822965; doi:10.1371/journal.pone.0340254)
Supplement: S1 Table — (PDF) [file pone.0340254.s001.pdf]

S1 Table Demographic Characteristics of Undergraduate Medical Students (N = 4494)

	Percentage	Number
**Gender**		
Male	35.34%	1588
Female	64.66%	2906
**Residence**		
Urban	45.46%	2043
Rural	54.54%	2451
**Ethnicity**		
Majority	58.59%	2633
Minority	41.41%	1861
**Grade**		
Grade 1	27.21%	1223
Grade 2	30.17%	1356
Grade 3	23.34%	1049
Grade 4	10.64%	478
Grade 5	8.63%	388
**Political Affiliation**		
Communist Party	5.30%	238
Communist Youth League	71.43%	3210
No Affiliation	23.28%	1046
**Family Economic Hardship**		
Yes	34.96%	1571
No	65.04%	2923
**Family Medical Background**		
Yes	29.57%	1329
No	70.43%	3165
**Mobile Internet Usage Intensity**		
<1h/day	6.25%	281
1-3h/day	32.53%	1462
3-5h/day	37.56%	1688
>5h/day	23.65%	1063
**Main Mobile Internet Usage Type**		
Educational & Professional Development	30.49%	1370
Social Networking	21.96%	987
E-commerce & Lifestyle Services	2.51%	113
Leisure & Entertainment	45.04%	2024
**Employment Intention** (Mean±SD)	3.15±1.28	
**Primary Care Employment Intention** (Mean±SD)	3.05±1.23	
