# Supplementary material for: The impact of mobile internet usage patterns on employment intentions of medical students: A cross-sectional study
Source: PLoS One. 2026 Jan 21;21(1):e0340254. doi: 10.1371/journal.pone.0340254 (PMC12822965; doi:10.1371/journal.pone.0340254)
Supplement: S2 Table — (PDF) [file pone.0340254.s002.pdf]

S2 Table Spearman Correlation Matrix for Variables Used in Regression Models

Variables	EI	PCEI	Mobile Internet Usage Intensity	Main Mobile Internet Usage Type	Gender	Residence	Ethnicity	Grade	Political Affiliation	Economic Hardship	Family Medical Bacngroun d
**EI**	1										
**PCEI**	0.6760	1									
**Mobile Internet Usage Intensity**	-0.0930	-0.129	1								
**Main Mobile Internet Usage Type**	-0.0585	-0.0481	0.0608	1							
**Gender**	-0.0339	-0.0358	0.0620	0.0632	1						
**Residence**	0.0812	0.0745	0.0451	-0.0054	0.0048	1					
**Ethnicity**	0.0644	0.0309	0.0015	0.0583	-0.0410	0.1750	1				
**Grade**	-0.0209	-0.0544	0.0139	-0.0446	-0.0162	0.0012	-0.0031	1			
**Political Affiliation**	0.0274	0.0178	0.0366	-0.0125	-0.0848	-0.0023	0.0308	-0.3690	1		
**Economic Hardship**	-0.0317	-0.0360	-0.0102	-0.0010	-0.0929	-0.2750	-0.0788	-0.0902	0.0370	1	
**Family Medical Bacnground**	0.0045	-0.0098	0.0285	-0.0584	0.0494	0.0566	0.0139	0.1170	0.1150	0.0178	1
